# The Massive Bleeding after the Operation of Hip Joint Surgery with the Acquired Haemorrhagic Coagulation Factor XIII(13) Deficiency: Two Case Reports

**DOI:** 10.1155/2013/473014

**Published:** 2013-02-28

**Authors:** Akio Kanda, Kazuo Kaneko, Osamu Obayashi, Atsuhiko Mogami

**Affiliations:** ^1^Department of Orthopaedic Surgery, Juntendo Shizuoka Hospital, Izunagaoka 1129, Izunokuni, Shizuoka 410-2295, Japan; ^2^Department of Orthopaedic Surgery, Juntendo University, Hongou 3-1-3, Bunkyo Tokyo 113-8431, Japan

## Abstract

Two women, aged 81 and 61, became haemorrhagic after surgery. Their previous surgeries were uneventful with no unexpected bleeding observed. Blood tests prior to the current surgeries indicated normal values including those related to coagulation. There were no problems with the current surgeries prior to leaving the operating room. At 3 hours after the surgery, the 81-year-old patient had an outflow of the drain at 1290 grams and her blood pressure decreased. She had disseminated intravascular coagulation (DIC). The 61-year-old woman had repeated haemorrhages after her current surgery for a long time. Their abnormal haemorrhages were caused by a deficiency of coagulation factor XIII(13). The mechanism of haemorrhagic coagulation factor XIII(13) deficiency is not understood, and it is a rare disorder. The only diagnostic method to detect this disorder is to measure factor XIII(13) activity in the blood. In this paper, we used Arabic and Roman numerals at the same time to avoid confusion of coagulation factor XIII(13) with coagulation factor VIII(8) that causes hemophilia A.

## 1. Introduction

Coagulation factor XIII(13) deficiency is a haemorrhagic disease based on decreased levels of factor XIII(13) [1]. The mechanism underlying factor XIII(13) deficiency is not known. Factor XIII(13) strengthens fibrin thrombi and furthermore protects the fibrin clot from enzymolysis and thus contributes to blood clot stabilization [2, 3]. Acquired haemorrhagic coagulation factor XIII(13) deficiency is classified into two categories based on an inhibitor of factor XIII(13) [1]. We experienced two rare cases of acquired coagulation factor XIII deficiency that did not involve the inhibitor. General coagulation tests, such as activated partial thromboplastin time (APTT) and prothrombin time international normalized ratio (PTINR), show almost normal values [4]. The only diagnostic method is the measurement of factor XIII(13) activity [1]. The only treatment is replenishment of factor XIII(13). The normal range of factor XIII(13) activity is generally 70% to 130% [1]. The coagulation factor activity level that distinguishes nonhaemorrhagic from haemorrhagic disease is not defined [5, 6]. Therefore, the targeted level of coagulation factor activity for treatment is not established [1].

## 2. Case Presentation

### 2.1. *Case*  1

**Figure 1:**
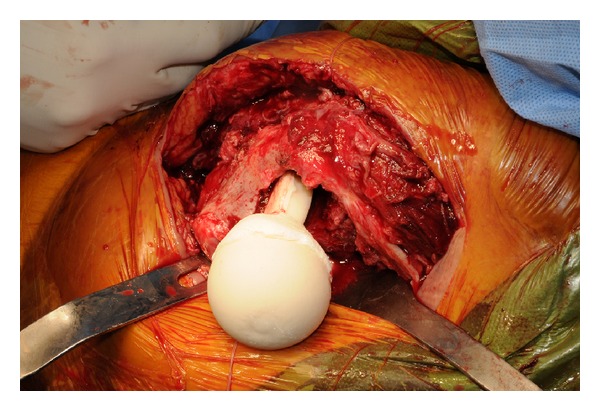
In the second operation, we removed the prosthesis, performed the irrigation, and inserted the cemented spacer mixed with the antibiotics for reducing the infection.

An 81-year-old woman had a total hip arthroplasty for osteoarthritis of the hip joint at a different hospital. Her coagulation tests showed almost normal values. Activated partial thromboplastin time (APTT) was 37.7 sec, and prothrombin time international normalized ratio (PTINR) was 1.20. The implant was inserted using a posterolateral approach. The operation lasted 97 minutes, and the total bleeding during the operation was 520 grams. The patient experienced no problems with the surgery before leaving the operating room. In her hospital room at 3 hours postoperative, however, the outflow from the drain was 1290 grams, her blood pressure decreased, and she had disseminated intravascular coagulation (DIC). She was given a blood transfusion and was treated for DIC. After a few days, she recovered from the DIC, but she developed an infection on the prosthesis. Based on culture tests, she was diagnosed as having a pseudomonas infection. At this point, she was transferred to our hospital for treatment of the infection. In our hospital, we noted that her surgical wound appeared red and swollen. Her blood tests showed C-reactive protein at 7.5 mg/dL and a white blood cell count at 9.8 10^3^/*μ*. Her coagulation tests did not show abnormal values with an activated partial thromboplastin time (APTT) at 31.2 sec and a prothrombin time international normalized ratio (PTINR) at 1.19. Since the infection continued and the risk of massive bleeding was low, we decided to remove the prosthesis to reduce the infection. At 41 days after the initial operation, a second operation was performed using a posterolateral approach (Figure 1). The operation lasted 145 minutes and the total bleeding during the operation was 1000 grams. She received a blood transfusion of 2 units RCC-LR. When leaving the operating room, her blood pressure was 125 over 60 mmHg, her heart rate was 132 per minute, and she was conscious. Thirty minutes after arriving at her hospital room, she suddenly had low blood pressure. Her systolic blood pressure was 50 mmHg, the amount of lost blood in the drain bag was 400 grams, and blood oozed from the surgical wound through the bandage. In her hospital room, she received a blood transfusion of 2 units RCC-LR and 2 units fresh frozen plasma. At postoperative three hours, blood still oozed heavily from her surgical wound. Her systolic blood pressure was 70 mmHg and her heart rate was 150 per minutes. Her blood tests indicated that hemoglobin was 4.0 g/dL. In the function blood coagulation tests, APTT was 122 sec, PTINR was 2.77, and FDP was 228 *μ*g/mL. Thus, she had disseminated intravascular coagulation (DIC) again. Because she had lost consciousness, we intubated her and used a mechanical ventilator. The next day, her hemoglobin was 9.2 g/dL and her blood platelet count was 8.9 10^4^/*μ*. In the function blood coagulation tests, APTT was 45.8 sec, PTINR was 1.85, and FDP was 284.1 *μ*g/mL. Her blood systolic pressure was 80 mmHg and her heart rate was 140 per minute. Since her condition did not improve, we gave her transfusions every day to stabilize her condition. However, we were not able to determine the cause of the bleeding based on her blood tests or on image diagnosis. At 44 days after the second operation, she resumed eating a normal diet since her general condition was improved. But the bleeding continued and the surgical wound was dehiscence and infected. By this time, she had received a total of 28 blood transfusions of RCC-LR, 8 units of fresh frozen plasma, and 20 units of platelet concentrate since the second operation. Unfortunately, we had not diagnosed the cause of the bleeding at this time. Because the surgical wound was dehiscence and the infection remained, we needed to perform a third operation on her. At 53 days after the second operation, we consulted a hematologist. He determined the cause of the bleeding as due to a decrease in coagulation factor XIII(13) and diagnosed her as having acquired coagulation factor XIII(13) deficiency. Her coagulation factor XIII(13) activity was 48% (normal value 70~140%). The treatment of acquired coagulation factor XIII(13) deficiency is replenishment of coagulation factor XIII(13) using fresh frozen plasma or a blood product. At day 65 after the second operation, we made plans for a third operation for irrigation and insertion of a cemented spacer mixed with antibiotics. As a countermeasure to the bleeding due to coagulation factor XIII(13) deficiency, we planned to give the blood product coagulation factor XIII(13) for five days after the surgery. At day 65 after the second operation, the third operation was performed using a posterolateral approach. The operation lasted 145 minutes, and the total bleeding during the operation was 470 grams. She had a blood transfusion of 4 units RCC-LR. When leaving the operation room, her blood pressure was 167 over 76 mmHg and her heart rate was 92 per minute. She was fully conscious. The amount of lost blood in the drain bag was 100 grams, and blood did not ooze from the wound through the bandage in the first 24 hours. Since the bleeding from the surgical wound was slight, we did not need to give an additional blood transfusion. Subsequently, she did not have low blood pressure and did not have signs of disseminated intravascular coagulation. In the third operation, no diastasis was noted in the surgical wound, and infection was absent. Her general condition stabilized, and she was transferred to the previous hospital for the next revision surgery. Unfortunately, a malignant tumor had developed in the pancreas and the planned surgery was cancelled.

### 2.2. *Case*  2

**Figure 2:**
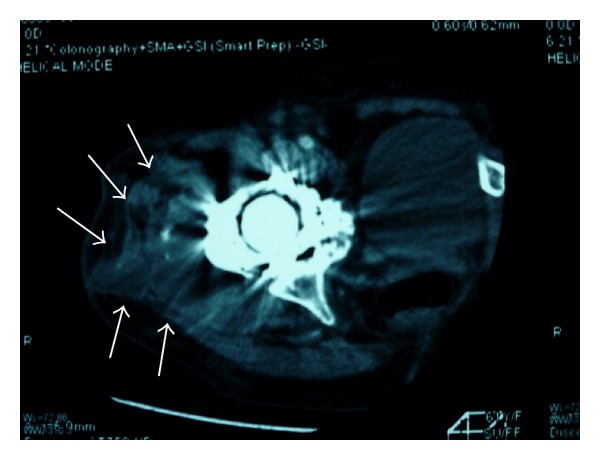
We tested for an arterial bleed with computed tomography using a contrast medium. Although active bleeding was not observed, a hematoma around the hip joint was visible in this test (white arrows).

**Figure 3:**
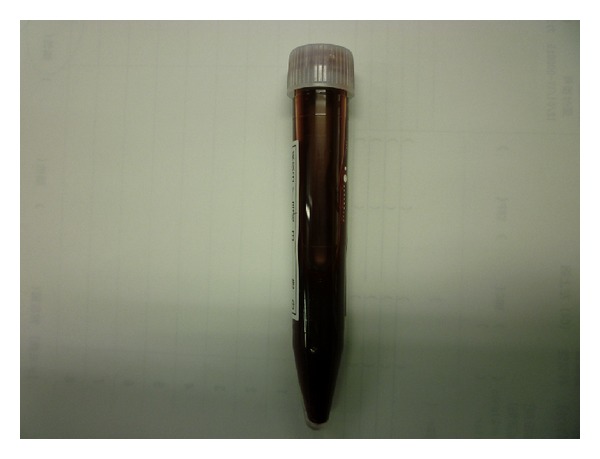
At postoperative day 27, we punctured the hematoma and 150 grams of uncoagulated bloody liquid was aspirated.

**Figure 4:**
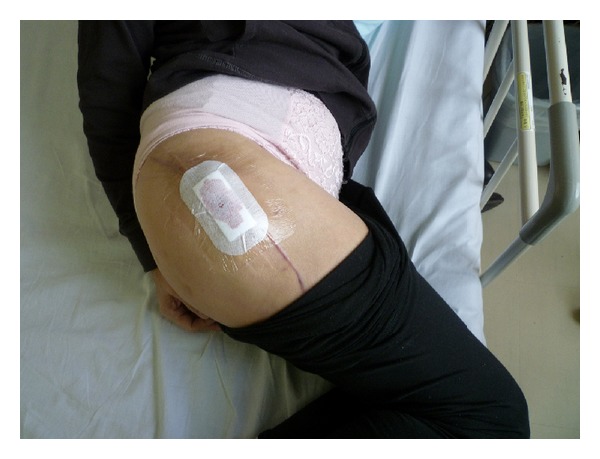
A physician at a rehabilitation hospital punctured the hematoma, releasing about 50 mL of uncoagulated bloody liquid. Subsequently bloody liquid continued to flow from the puncture needle hole.

**Figure 5:**
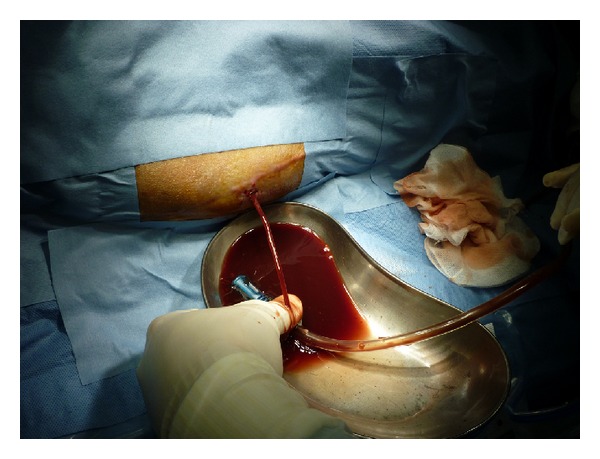
At postoperative day 115, we completely removed the hematoma via approximately a 2 cm surgical skin incision. We removed about 1,000 grams of uncoagulated lightly colored bloody liquid.

**Figure 6:**
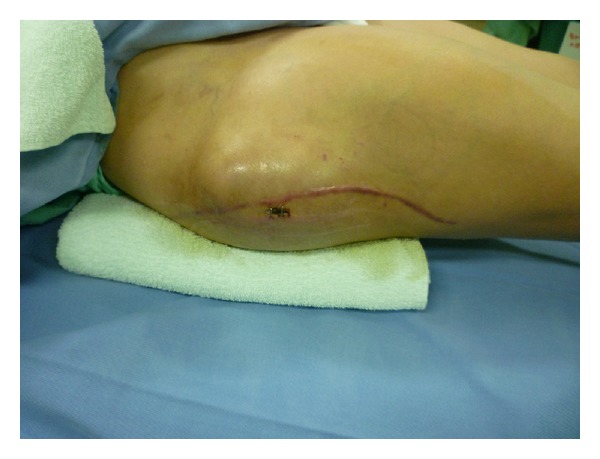
At day 3 after complete removal of the hematoma, the hematoma enlarged gradually and the thigh pain returned.

A 61-year-old woman had the third operation of revision hip arthroplasty for displacement of the cup from a previous total hip arthroplasty that had been performed when she was 43 years old. The previous implant was removed and a new implant was inserted through a posterolateral approach. In her previous two surgeries, there were no problems with unexpected bleeding. In the blood tests before this current operation, there were no abnormal values including coagulation tests: APTT was 27.1 sec, and PTINR was 0.87. The operation lasted 500 minutes and the total bleeding during the surgery was 3060 grams. Before leaving the operating room, no problems related to the surgery were noted. In her hospital room, the outflow from the drain was 1110 grams at the postoperative time of 8 hours. Her blood pressure did not decrease, and she did not have DIC. At postoperative day 3, an outflow from the drain was 250 grams, and thus we decided to remove the drain. After removing the drain tube, the surgical wound was not inflamed, the patient was not in pain, and her blood tests showed normal values. But, at postoperative day 7, suddenly, she complained of severe thigh pain and her thigh was swollen. We suspected that postoperative bleeding had continued, but her blood hemoglobin was 6.7 g/dL which was almost the same value as her previous test. Based on these findings, we did not provide any treatment. At postoperative day 18, her thigh pain naturally disappeared. At postoperative days 23 and 27, she complained of severe thigh pain and her thigh was swollen again. We thought that the cause of the thigh pain and swelling was arterial bleeding and tested for this complication with computed tomography using a contrast medium. Active bleeding was not observed; however, a hematoma around the hip joint was visualized by this method (Figure 2). Thus, we punctured the hematoma and 150 grams of uncoagulated bloody liquid was aspirated (Figure 3). Because the thigh pain was gradually relieved, we did not do any further treatment. Her thigh remained slightly swollen. She was transferred to a rehabilitation hospital. At the rehabilitation hospital, the hematoma enlarged gradually and she again complained of thigh pain. Therefore, a doctor at the rehabilitation hospital punctured the hematoma and about 50 mL of uncoagulated bloody liquid was aspirated. Because the bloody liquid continued to flow from the puncture needle hole, she was admitted back to our hospital (Figure 4). We still thought that the cause of the thigh pain and swelling was an arterial bleed, but this was not observed by computed tomography using a contrast medium. We planned to perform angiography to see if she had a transcatheter arterial embolization. Before the angiography at postoperative day 115, we completely removed the hematoma with a 2 cm surgical skin incision. We removed about 1,000 grams of uncoagulated lightly colored bloody liquid (Figure 5). During the surgery, it is easy to confirm internal bleeding, but we did not find any active bleeding. Three days later, the hematoma had enlarged gradually and the thigh pain returned (Figure 6). In light of the previous case, we suspected acquired coagulation factor XIII(13) deficiency and tested factor XIII(13) activity. Coagulation factor XIII(13) activity was 49% (normal value 70%~140%). As a countermeasure to the bleeding due to coagulation factor XIII(13) deficiency, we decided to give her coagulation factor XIII(13) for five days after surgical removal of the hematoma. We completely removed the hematoma via the same 2 cm surgical skin incision. Five days later, the hematoma enlarged slightly, but she did not have thigh pain. Thus, after surgery she had a blood transfusion of 12 units fresh frozen plasma in all for three days after she had the blood product factor XIII(13) for five days. At the time when administration of blood product was completed, her factor XIII(13) activity was 200% (normal value 70%~140%). Because the hematoma did not enlarge and she did not have thigh pain, she left the hospital and went home. Until now, we have not seen a recurrence of the hematoma.

## 3. Discussion

The mechanism of coagulation factor XIII(13) deficiency is unknown. Coagulation factor XIII(13) deficiency is a haemorrhagic disease based on a decrease of factor XIII(13) [1]. Factor XIII(13) is a heterotetramer composed of two-type A and two-type B subunits [7]. It is activated by thrombin, and the activated form cross-links fibrin for clot formation. Coagulation factor XIII(13) strengthens fibrin thrombi and furthermore protects the fibrin clot from enzymolysis. By these actions, factor XIII(13) contributes to blood clot stabilization [2, 3]. Factor XIII(13) deficiency is classified as either a congenital or an acquired disease [1]. There are only several hundred cases in the world reported documenting this deficiency. The main symptoms are haemorrhage, abnormal wound healing, and recurrent abortion. The severe form involving omphalorrhagia is apparent within a few days after birth. The principal cause is a genetic defect in factor XIII(13), and the treatment of this disorder is replacement therapy with factor XIII(13) [8]. Acquired factor XIII(13) deficiency is classified into two categories based on the presence of an inhibitor of factor XIII(13) [1]. The inhibitor of factor XIII(13) can occur in patients with an autoimmune disease, cancer, and those taking certain drugs, but the cause in half of the patients is unidentified [1, 9]. The normal range of factor XIII(13) activity is generally 70% to 130% [1]. The coagulation factor activity level that defines nonhemorrhagic or hemorrhagic is variable depending on the patient [5, 6]. Therefore, the targeted level of the coagulation factor activity for treatment is not established [1]. The treatment is replenishment of factor XIII(13), with simultaneous immunosuppressive therapy using an adrenocorticosteroid or a cyclophosphamide. Furthermore, either plasma exchange therapy, adsorptive therapy for the antibody, or immunoglobulin high dose therapy is also effective [1]. When the inhibitor for coagulation factor XIII(13) is not present, such as when the factor XIII(13) decrease is caused by degradation, the treatment is replenishment of factor XIII(13) [1]. When factor XIII(13) is not available immediately fresh frozen plasma is used [10]. Although the active level of factor XIII(13) for normal hemostasis is not established, it is important to try to achieve an activity level of factor XIII(13) in the normal range for those with serious bleeding [1]. With orthopedic surgery, especially artificial joint replacement, infections are more likely to occur due to hematomas from postoperative haemorrhage. Actually, case  1 patient developed the wound infection after the hematoma formed. As in this case, patients may have no bleeding issues with prior surgeries or even during the surgery but then suddenly have bleeding in the postoperative period. When a preoperative blood test shows normal values and then postoperative aberrant haemorrhage, occurs this deficiency should be suspected, and the activity of coagulation factor XIII(13) should be examined. Acquired coagulation factor XIII(13) deficiency is a rare bleeding disorder, but we experienced two cases by chance. 

We experienced two rare cases of acquired coagulation factor XIII(13) deficiency. The mechanism of the haemorrhagic coagulation factor XIII(13) deficiency is unknown. When preoperative blood tests show normal blood values and aberrant haemorrhage occurs postoperatively, one should suspect this disease and examine the activity of coagulation factor XIII(13).
